# Cooperation & Liaison between Universities & Editors (CLUE): recommendations on best practice

**DOI:** 10.1186/s41073-021-00109-3

**Published:** 2021-04-15

**Authors:** Elizabeth Wager, Sabine Kleinert, on behalf of the CLUE Working Group, Volker Bähr, Ksenija Bazdaric, Michael Farthing, Michele Garfinkel, Chris Graf, Zoë Hammatt, Lyn Horn, Susan King, Debra Parrish, Bernd Pulverer, Paul Taylor, Gerrit van Meer

**Affiliations:** 1 Sideview, 19 Station Road, Princes Risborough, HP27 9DE UK; 2 The Lancet, London, UK

**Keywords:** Research integrity, Research institutions, Journals, Misconduct, Research reporting, Peer review, Fabrication, Falsification, Plagiarism

## Abstract

**Background:**

Inaccurate, false or incomplete research publications may mislead readers including researchers and decision-makers. It is therefore important that such problems are identified and rectified promptly. This usually involves collaboration between the research institutions and academic journals involved, but these interactions can be problematic.

**Methods:**

These recommendations were developed following discussions at World Conferences on Research Integrity in 2013 and 2017, and at a specially convened 3-day workshop in 2016 involving participants from 7 countries with expertise in publication ethics and research integrity. The recommendations aim to address issues surrounding cooperation and liaison between institutions (e.g. universities) and journals about possible and actual problems with the integrity of reported research arising before and after publication.

**Results:**

The main recommendations are that research institutions should:

1. develop mechanisms for assessing the integrity of reported research (if concerns are raised) that are distinct from processes to determine whether individual researchers have committed misconduct;
2. release relevant sections of reports of research integrity or misconduct investigations to all journals that have published research that was investigated;
3. take responsibility for research performed under their auspices regardless of whether the researcher still works at that institution or how long ago the work was done;
4. work with funders to ensure essential research data is retained for at least 10 years.

Journals should:

1. respond to institutions about research integrity cases in a timely manner;
2. have criteria for determining whether, and what type of, information and evidence relating to the integrity of research reports should be passed on to institutions;
3. pass on research integrity concerns to institutions, regardless of whether they intend to accept the work for publication;
4. retain peer review records for at least 10 years to enable the investigation of peer review manipulation or other inappropriate behaviour by authors or reviewers.

**Conclusions:**

Various difficulties can prevent effective cooperation between academic journals and research institutions about research integrity concerns and hinder the correction of the research record if problems are discovered. While the issues and their solutions may vary across different settings, we encourage research institutions, journals and funders to consider how they might improve future collaboration and cooperation on research integrity cases.

**Supplementary Information:**

The online version contains supplementary material available at 10.1186/s41073-021-00109-3.

## Background

Inaccurate, false or incomplete research publications may mislead readers. This is potentially harmful because decisions on future research, public policies and medical treatments are often based on academic articles. It is therefore important that any problems are identified and rectified promptly. This usually involves collaboration and the sharing of information between the research institutions and academic journals involved.

Journals and research institutions (e.g. universities) share common interests when concerns arise over the integrity of research reports that are submitted for publication or are published. Concerns about the integrity of reported research may arise after publication or during editorial assessment or peer review, or from pre-publication screening (e.g. for plagiarism or image manipulation); therefore journals may be the first to suspect problems. However, journals usually do not have all the evidence, or a specific mandate, to conduct a formal investigation. Therefore it is important for them to alert the relevant institution(s) and funder(s). Liaison between institutions and journals is also important after an institutional investigation, especially if the investigation indicates problems with published work (whether or not this is deemed to have been caused by misconduct), so that the research record can be corrected. However, cooperation between journals and research institutions about research integrity concerns is not always straightforward and both report difficulties and frustrations.

In 2012, the Committee on Publication Ethics (COPE) published guidelines on cooperation between research institutions and journals on research integrity cases [1]. These guidelines were discussed by participants at a Focus Track session at the 3rd World Conference on Research Integrity (WCRI) in Montreal in 2013 and a series of questions was formulated on which further guidance was desired [2].

## Methods

In July 2016, a 3-day workshop was held in Heidelberg at the European Molecular Biology Organization (EMBO). Its aim was to address the questions raised in Montreal [2], to understand the reasons why communication and cooperation between journals and institutions is sometimes challenging, and to identify practical solutions to problems. The meeting was convened by the authors (Elizabeth Wager and Sabine Kleinert) and Michele Garfinkel (of EMBO).

The meeting brought together an invited group of editors and publishers of scholarly journals, people working at universities and national research integrity organizations (including research integrity officers, a university vice-chancellor and a dean), a lawyer with experience of representing researchers, journals and universities in research misconduct cases, and policy experts (the CLUE Working Group – see listing at end of article). It provided an opportunity for participants to discuss their expectations of each other and their experiences of relevant cases involving interactions between research institutions and journals. The 14 members of the CLUE Working Group were selected for their broad knowledge of publication ethics and research integrity in a range of settings (e.g. large and small institutions and journals) and came from Australia, Croatia, Germany, the Netherlands, South Africa, UK, and USA (to ensure different legislative systems were represented). The discussions in Heidelberg were structured around the issues raised at WCRI in Montreal which had been published and circulated before the meeting [2]. Seven of the 14 Working Group members had also contributed to the Montreal report.

The Heidelberg workshop was structured to ensure all topics identified in Montreal and the earlier COPE guidelines were addressed (see Agenda in supplementary material). The meeting adopted structured discussion and note-taking rather than a formal decision-making process (eg Delphi). Wherever possible, consensus was achieved on all proposed recommendations at the meeting. Where differences of opinion persisted, this was noted. After extensive and detailed discussion at the 3-day meeting, an initial draft was prepared by the authors and circulated for review by the Working Group allowing further discussion of the text and recommendations (via email and phone calls). A revised draft was then approved by the Working Group and posted as a preprint for wider comment [3]. It was also discussed at a session of the 5th WCRI in Amsterdam in 2017. All comments received after the workshop related to the predefined issues and proposed recommendations (i.e. no additional issues were identified at this stage). Comments posted on the preprint and made at WCRI were collated and circulated by email for further discussion by the Working Group who agreed the final version of the text submitted to the journal and the revised version in response to peer-reviewers’ suggestions.

### Scope

These recommendations cover interactions between representatives of scholarly, peer-reviewed journals and research institutions about cases in which there are concerns about the integrity of reported research that has been submitted for publication to the journal whether or not it has been (or will be) published. While the recommendations focus on universities and journals, possible roles for other bodies such as major funders and national (or regional) research integrity organizations are also addressed.

### Terminology and definitions

In this document, the term ‘journal’ is used to mean editors and publishing staff who handle cases or develop policy on research and publication integrity. The acronym CLUE (standing for Cooperation and Liaison between Universities and Editors) uses the term ‘universities’ to include all types of research institution (mainly focusing on academic institutions) and ‘editors’ to refer to all journal representatives.

Research integrity describes ‘the principles and standards that … ensure validity and trustworthiness of research’ [4]. This broad terms encompasses all aspects of responsible research and reporting practices. Concerns about research integrity (which, individually may be referred to as cases) do not necessarily imply misconduct but may include deficiencies in study design or analytical methods as well as other types of ‘honest’ error. As noted in the COPE guidelines, journals have responsibility for the trustworthiness (or soundness) of what they publish but this does not always align with institutions’ definitions of research misconduct [1]. In other words, it is possible for research reports to be misleading and therefore to require correction or retraction even when the authors/researchers are not considered to have committed research misconduct by their institution. This document therefore covers any deficiencies in the integrity of research reports which means that they, for whatever reason, require correction or amendment, or should not remain on, or become part of, the research record.

Another distinction which may be more important to institutions than to journal editors (and readers) is whether problems are associated with the underlying research or with its reporting. A partial or inaccurate report of properly-conducted research may mislead readers just as much as a report of poorly-conducted or fraudulent work. Research integrity also encompasses attribution of research and publications (i.e. authorship and plagiarism) which are not directly related to the quality of the research. To save repeated references to ‘the research and/or its reporting or attribution’ we use the term ‘reported research’ and ‘research reports’ to cover all these aspects.

The terms ‘inquiry’ and ‘investigation’ refer to formal processes conducted by research institutions to determine whether a researcher/employee has committed misconduct. One of the issues discussed at the CLUE meeting was the extent to which journals should assess evidence of misconduct. While it was agreed that it is not the role of journals to conduct formal research misconduct investigations, we recognise that, in some cases, it may be appropriate for journals to consider evidence relating to the integrity of a publication or submission. Institutional investigations tend to focus on the guilt or otherwise of the researcher(s) concerned and seek to determine whether their behaviour amounts to research misconduct, however that is defined. However, journals are more concerned with determining whether the reported research has integrity (i.e. can be trusted, is properly reported and attributed, and is reliable). These are different questions that are answered in different ways and carry different obligations. Journals may conduct their own assessments of the integrity of the research reported in a manuscript or article, but such assessments are often limited by the journal’s access to necessary information. Institutional consideration may focus on the behaviour or motivations of the individual researcher(s) but may not fully address the broader questions of research and reporting integrity, such as methodological weaknesses, that the journal needs to be answered.

## Results

The parallel session at WCRI in Montreal in 2013 identified 36 questions or challenges [2]. These were grouped under the headings: (1) When editors have concerns about research or publication integrity; (2) When institutions receive evidence or questions about research or publication integrity; (3) When institutions investigate suspected misconduct; (4) Role of funders and other bodies; (5) What can journals, institutions and funders do to prevent and detect misconduct and errors? The Workshop held in Heidelberg in 2016 was divided into 7 sessions on the themes of: institutions’ expectations of journals and (separately) journals’ expectations of institutions in cases of (i) suspected author or reviewer misconduct, (ii) proven misconduct, and (iii) other situations, with a final session focusing on other stakeholders (e.g. funders, journalists, lawyers, research integrity organizations). Following the meeting and discussion of a draft report, 14 key issues were identified and these were later organized into three themes. The issues are presented below, with a brief summary of the discussion and recommendations (except for Issues 3 and 8 where the group felt unable to make a universal recommendation because of the differences in laws and practices between countries).

### Recommendations on best practice

#### (I) When journals suspect problems with submitted work

##### Issue 1: journals often have difficulty identifying somebody with responsibility for research integrity at an institution

In many areas outside North America, universities either do not have a research integrity officer or office (RIO), or the person or department with responsibility for research integrity (and their contact details) is not clearly identified on the institution’s website [5]. Identifying the right contact person is also difficult because various titles are used for this function.

If the corresponding author’s institution does not have a RIO, the authors may identify a suitable person at any of their institutions. If no such person can be identified at any of the institutions involved with the research, the authors should be asked to nominate a senior faculty member (e.g. dean or pro-vice chancellor with responsibility for research, or the chair of the research ethics committee or institutional review board) who was not directly involved with the research (and is not an author) who could be contacted if the journal has any concerns about research integrity.

Requiring researchers to provide contact details of a person with responsibility for handling research integrity allegations at their institution should not only enable journals to contact this person if concerns arise, but may raise awareness of RIOs among researchers and encourage institutions to make such an appointment and publish their contact details prominently on institutional websites. Details of the contact person for research integrity enquiries should not be published by the journal, but should be retained, should the need arise to contact them.

*Recommendations*

Institutions should have a research integrity officer (or office) and publish their contact details. National research integrity bodies (or other appropriate organizations) should keep a register of people with responsibility for handling research integrity allegations at their country’s institutions, to enable journal editors (and others) to contact them.

Where such lists are not available, journals should request corresponding authors to provide the name and email address (or telephone number) of their institution’s RIO (or of an individual with responsibility for handling research integrity allegations) when submitting the article.

##### Issue 2: what should journals do when reviewers say findings look ‘too good to be true’ in the absence of specific evidence?

If a peer reviewer raises a concern about the integrity of research, especially if s/he suggests that the results are ‘too good to be true’, the journal should ask the reviewer for more details (e.g. to explain why they gave this opinion) and may consider alerting the institution to concerns of fabrication or falsification if they consider they are well founded. Journals therefore need to determine whether to contact an institution and, if so, what information they should share, and whether this should be done before discussing concerns with the authors (see Issue 4).

Peer reviewer reports and comments to the editor should generally only be shared with authors’ institutions with the reviewers’ express permission. Similarly, the identity of the peer reviewer should not normally be revealed to the authors’ institutions in cases of suspected problems with submitted or published work (unless the reviewer has agreed to this or the journal publishes signed reviewer comments in an open peer review system).

It is helpful for journals to share suspicions about the integrity of reported research with institutions (as well as more specific concerns or clear evidence of problems) because institutions are able, and have a duty, to assess concerns about possible integrity breaches such as data fabrication or falsification by researchers. Another reason why journals should raise concerns about reported research is that the institution should have a more complete picture of the researcher’s behaviour than the journal (which usually has information only from one article), and such evidence may be important to trigger or inform an investigation. Sophisticated data fabrication or falsification may only become obvious when several publications are assessed, or when raw data or other forensic evidence are available [6]. Therefore, in such cases, while individual journals may have some suspicions, the full picture is usually available only to the institution. Furthermore, alerting an institution may prevent the research from being submitted to other journals (which would be unaware of the first journal’s concerns) before it has been properly assessed.

*Recommendations*

Journals should develop criteria for determining whether, and what type of, information should be passed on to institutions.

When journals share evidence relating to possible research integrity problems with institutions the identity of peer reviewers or other people raising concerns should not be revealed (unless this is already published or the individuals have given permission for this disclosure).

In addition to sharing any direct evidence of plagiarism, fabrication or falsification with institutions, journals should develop criteria for determining when reviewer or editor suspicions that work is ‘too good to be true’ or that something is ‘not right’ should be shared.

##### Issue 3: journals do not know whether an informal ‘off the record’ approach to an institution is possible

Since journals are typically not in a position (either legally or practically) to conduct detailed, formal investigations it is not always possible for an editor to obtain clear evidence or to judge whether an allegation is well-founded on the basis of submitted or published reports. While journals may request source data from authors, they do not have legal powers to obtain this, nor do they have access to laboratory notebooks or equipment logs, or the possibility to interview staff. Therefore, since journals do not normally have access to all the relevant information, their peer reviewers and editors may only be able to indicate they suspect that something is wrong.

Therefore, journals may sometimes want to contact institutions informally, to discuss their suspicions or concerns, or raise non-specific allegations about research integrity, without necessarily invoking a full investigation. Journals may also wish to know whether a researcher is currently being, or has recently been, investigated for suspected misconduct.

Journals need to understand that in some jurisdictions (for example, the United States) such an ‘off the record’ discussion is not always possible, as institutional research integrity officers and all those involved with investigations have to maintain the maximum confidentiality possible until the formal process has concluded and such conversations must be documented as part of the institutional record. Institutions risk being sued if they breach this confidentiality, e.g. by revealing that a researcher is under investigation. However, in other regions, the situation is different and it may be possible to discuss concerns informally and for universities to disclose whether an individual is currently under investigation.

##### Issue 4: should journals always contact authors about research integrity concerns?

In most cases, when journals have concerns about the integrity of submitted or published work, they should first raise them with the authors (usually starting with the corresponding author). This allows researchers to provide clarification, explanation or further information. Contacting authors is considered to reflect ‘due process’ or procedural fairness, and avoids wasting institutional and editorial time and resources over issues that arise from honest error and can be handled in a straightforward way by the journal. When approaching authors, journals are advised to describe concerns using neutral rather than accusatory language, for example highlighting the amount of text similarity rather than accusing an author of plagiarism. The presumption, at this stage, is that the authors are ‘innocent until proven otherwise’.

However, journals should be aware that in cases of suspected data fabrication or falsification, raising concerns with the authors first could enable researchers to destroy or alter evidence that might be important for an institutional investigation (http://retractionwatch.com/2014/07/22/accounting-professor-faked-data-for-two-studies-destroyed-evidence-university-report/, https://www.baruch.cuny.edu/rio/research_misconduct_examples.htm). Therefore, when journals have well-founded suspicions or evidence of falsification or fabrication they should consider informing the institution at the same time as, or before, they contact the author(s).

Such cases are likely to be rare, since the circumstances in which journals have access to raw data are currently limited (but may include Western blots and other images). This situation may change as publication of research data becomes more widespread (http://aims.fao.org/activity/blog/%E2%80%98-state-open-data%E2%80%99-figshare%E2%80%99s-report-global-trends-around-open-data).

If a journal discovers evidence of falsification (e.g. inappropriate manipulation of images) or major plagiarism (e.g. reports from text-matching software verified by an editor) the journal should retain the evidence and should offer to share it with the institution. (See, for example the EMBO Press classification for image aberrations [7].) However, care should be taken to avoid revealing the identity of peer reviewers, or other people raising concerns, to an institution against their wishes or without their permission. Ensuring the anonymity of internal whistleblowers (i.e. members of a research group or department who raise concerns about colleagues or collaborators) may be difficult since, even if their name is not revealed, the source may be obvious to the authors if only a few people know about certain details of the research.

*Recommendation*

Journals should develop criteria to determine when the authors’ institution(s) should be contacted immediately without (or at the same time as) alerting the author(s). This would occur only in exceptional cases when journals have evidence of substantive or significant falsification or fabrication of data.

##### Issue 5: if a journal suspects that an author or peer reviewer has failed to disclose a relevant competing interest, should they refer this to the institution?

Readers, authors or reviewers sometimes suggest that relevant competing interests have not been disclosed during the review process or in a publication. If such allegations or concerns cannot be resolved after discussion with the authors or reviewers concerned (e.g. by publishing a correction if information has been omitted from a publication, or seeking additional peer review), the journal may consider contacting an institution. However, institutional responses vary. Some institutions maintain lists of researchers’ current interests and have policies about disclosure of competing interests. In such cases, it is appropriate for journals to raise concerns with the institution and to ask them for relevant information. However, not all institutions register such information, and, if they do not, they may be unable to respond to the journal’s enquiries. While failure to disclose a relevant interest is not always categorised as research misconduct, it is generally recognised to be poor practice and usually requires action by the journal (which will depend on the severity of the case). The WCRI notes that ‘being transparent about and managing competing interests’ is one aspect of research integrity [4].

*Recommendation*

Institutions and funders should be responsive to journal requests for information to ensure that peer reviewers’ and authors’ competing interests are properly disclosed.

##### Issue 6: if a journal rejects an article about which either reviewers or editors have raised concerns about integrity, authors may simply submit it to another journal, perhaps after concealing problems more effectively

The COPE Code of Conduct for journal editors noted that ‘Editors should not simply reject papers that raise concerns about possible misconduct. They are ethically obliged to pursue alleged cases.’ [8] In other words, journals should seek explanations from authors even if they do not intend to accept their publication and should contact institutions, if required, regardless of publication status.

One suggestion made at the meeting was for each institution to maintain a repository of submitted manuscripts. Researchers affiliated to an institution would be expected to send a copy of all submissions to this repository. These would not be made public but the database could be used to check the history of a publication and document any changes made by authors (e.g. when submitting to a different journal after a rejection). Such a database of submitted manuscripts would be useful for institutional investigations and would permit assessment of all of a researcher’s work. To be workable this process would need to be straightforward and not excessively burdensome on researchers.

*Recommendation*

Journals should seek explanations from authors about research integrity concerns even if they do not intend to accept their publication and should inform institutions, if appropriate, regardless of publication status.

#### II. Investigating problems with research and its publication

##### Issue 7: institutional focus on strict definitions of research misconduct may hamper communication about broader issues of research integrity

Journals have a responsibility to correct or retract any publications that give misleading accounts of research methods, findings, analyses or authorship, regardless of whether this is determined to have been due (or related) to misconduct or to error. However, many institutions and research integrity bodies focus solely on determining narrowly defined misconduct and establishing the burden of proof for each particular case. Furthermore, definitions of misconduct vary between jurisdictions. For example, the US Office of Research Integrity (ORI) considers only cases of fabrication, falsification or plagiarism (FFP) in research funded by the US Public Health Service [9] while the Australian Code for the Responsible Conduct of Research takes a more inclusive approach [10].

Because of the possible serious consequences of a misconduct finding for individuals and institutions, and the importance of conducting rigorous and fair proceedings (and the costs associated with these), thresholds for launching a full inquiry or investigation may be high. This may give journals the impression that institutions are reluctant to cooperate or respond to their enquiries.

It would therefore be helpful if institutions had mechanisms for assessing the integrity of reported research in response to concerns raised by journals or others. The focus of such assessment should be solely on determining the integrity of the research itself, and its reporting (in other words, the likelihood that journal readers may be misled or confused), rather than on the behaviour or intentions of the researchers. Such assessments should permit institutions to respond more rapidly to journal enquiries and without concerns about breaching confidentiality related to institutional policies or employment processes. However, such assessments would not prevent further investigation through the institution’s established processes for handling misconduct allegations.

*Recommendation*

Institutions should develop mechanisms for assessing the integrity of research reports that are submitted to, or published by, academic journals, which could be used if concerns are raised; these mechanisms should be distinct from processes to determine whether misconduct has occurred.

##### Issue 8: institutions may feel legally bound to keep disciplinary hearings confidential and may therefore feel unable to communicate or share details of on-going investigations with journals

Journals have a responsibility to alert readers to published material that may be misleading (for whatever reason). Even when misleading research does not cause direct public harm, it may lead to the waste of other researchers’ time and resources. The need for journals to alert readers promptly to potentially misleading articles is especially great in applied research since decisions affecting individuals and public policies may be based on publications. Journals may therefore wish to know if an investigation has been started, and may wish to alert readers before an investigation (and appeal process) has concluded (e.g. by an expression of concern).

However, in many jurisdictions, research misconduct investigations and disciplinary hearings are considered confidential and institutions/employers may therefore feel unable to share details with journals. This approach may prevent journals from fulfilling their responsibilities to their readers, for example by publishing an expression of concern. Journals need to be aware that practices vary between countries. For example, the European Network of Research Integrity Offices (ENRIO) notes ‘when it comes to the level of transparency and confidentiality regarding investigations, traditions and legislative mandates vary across Europe’ [11].

Various solutions to this problem were discussed at the CLUE meeting. One suggestion was for journals to require authors to disclose any investigations or proceedings and thus waive the confidentiality accorded by law within their contract with the journal.

Another suggestion was that researchers’ employment contracts should specify that, in cases of suspected or proven misconduct, harm to research participants, or other circumstances affecting the validity of a research report, the employees’ usual right to confidentiality in disciplinary proceedings would be waived to allow the institution to communicate relevant details to the journal and other parties. The CLUE meeting participants recognised that such solutions might be hard for journals to enforce, or may require changes in employment legislation, and therefore put them forward for discussion rather than as recommendations.

##### Issue 9: journals and institutions may be asked about publications relating to research that took place many years ago

While investigation of historical research may pose more challenges than inquiries into more recent work, concerns should not be dismissed solely on the grounds that the research was done a long time ago. If plausible evidence of serious problems is raised, it should, ideally, be examined, regardless of when the problems occurred. However, since the difficulties of contacting authors and accessing original data increase over time, it is reasonable for journals and institutions to prioritise the investigation of recent over historical work.

Institutions should take responsibility for research performed under their auspices regardless of whether the researcher still works at that institution. Even if a researcher has moved to another institution, or has retired, the appropriate investigation should take place.

Investigations into the work of researchers who have died, are chronically incapacitated or have left research altogether, is especially difficult. However, in such cases, institutions should make their best efforts to establish whether the work has integrity, so that journals can determine whether readers should be alerted to concerns. Although probably a rare occurrence, this is another situation in which assessing the integrity of findings and reports needs to be separated from determining whether misconduct was committed by an individual researcher.

It is often impossible for an institution to investigate the integrity of a piece of research and its reporting without access to the source data. This can be problematic if data are not retained, as suspicions sometimes emerge several years after publication [6]. Also, if data are kept only by individual researchers, files may be lost unintentionally or deliberately destroyed or altered.

Permanent, public deposition of data is the ideal, because it allows immediate scrutiny by anybody interested, which may reveal errors or misconduct. However public posting of individuals’ personal or clinical data may not be possible due to the need for confidentiality (e.g. of medical records).

We encourage institutions and funders to review current data retention standards which may prevent effective investigation of historical data (e.g. we suggest that the 6-year period required for the retention of personal health data in the US under the Health Insurance Portability and Accountability Act (HIPAA) [12, 13] is too short). We also encourage debate on the risks and benefits of conventions in certain disciplines of destroying sensitive data, such as interview transcripts, to protect the confidentiality of research participants and to develop alternative systems (e.g. locked, secure deposition) to permit later investigation, if required.

*Recommendations*

Institutions should take responsibility for research performed under their auspices regardless of whether the researcher still works at that institution or how long ago the work was done.

Research institutions and major funders should ensure that essential research data are retained for at least 10 years (enabling the validation of published findings). Responsibility for data storage (e.g. for multicentre studies) should be defined in funding agreements.

##### Issue 10: concerns may be raised about research that involved several institutions

When research involves several institutions, one of the institutions usually takes a primary or coordinating role in relation to the funding. This primary institution should be the initial point of contact and take the lead in responding to concerns about research integrity. Ideally, research agreements should specify this and also set out responsibilities for data deposition and retention [14].

The International Committee of Medical Journal Editors (ICMJE) states that authors should be accountable for answering questions about research and identifying which author was responsible for each aspect if questions arise [15]. We suggest extending this guidance so that authors are also expected to identify where each component of a project was done, and therefore which institution should be responsible for investigating any concerns about it.

*Recommendation*

Research agreements should specify the primary institution that will coordinate the response to concerns about research integrity (following liaison with the other institutions, as required).

##### Issue 11: journals and institutions may be contacted by whistleblowers who conceal their identity, use pseudonyms or request anonymity

Allegations and concerns about the integrity of research and its reporting should be taken seriously by journals and institutions regardless of their source. Institutions should have policies about whistleblower protection and about the handling of cases from anonymous whistleblowers. Such allegations should be considered on their merits rather than being dismissed automatically. Therefore, an individual’s refusal to reveal their name, use of a pseudonym, or request to remain anonymous, should not prevent either a journal or an institution from taking allegations seriously. However, both journals and institutions need reassurance that an allegation is well-founded and is not simply a personal vendetta. Journals and institutions may therefore request further details or information from the correspondent and, if evidence of a problem is not forthcoming, it is reasonable for journals not to raise the concern with the university or for an institution to decide not to proceed with an inquiry or full investigation. However, this is a matter of judgement for both journals and universities (which should apply to both named and anonymous complainants), so we recommend a flexible approach, depending on the seriousness of the alleged problem or behaviour and the plausibility of the evidence provided. Journals should not feel compelled to respond to vexatious complaints.

*Recommendation*

Anonymous or pseudonymous allegations about research integrity made to journals or institutions should be judged on their merit and not dismissed automatically.

##### Issue 12: who should investigate if a peer reviewer is suspected of acting inappropriately?

Universities should recognize peer review as a legitimate part of research and academic activity and should encourage accountable and responsible behaviour from their researchers when they act as reviewers or editors [16]. However, even when peer review is viewed as part of general academic duties, the reviewer’s institution may not be equipped to investigate suspicions of reviewer misconduct since most of the relevant information will be held by the journal. In such situations, the journal may therefore have to initiate its own investigation, following the COPE flowchart about how to handle cases of suspected reviewer misconduct [17]. Since investigation of peer review manipulation requires access to journals’ editorial records, publishers should ensure these are retained for similar time periods as research records. Evidence of serious misconduct by researchers acting as peer reviewers (e.g. stealing ideas or material from the articles they were invited to review) should be shared with their institution. Therefore, journals should inform reviewers that their identity might be disclosed to their institution in cases of suspected misconduct and that possible serious misconduct will be addressed by the institution.

*Recommendation*

Journals should share evidence of misconduct by researchers acting as peer reviewers with their institution and follow the relevant COPE flowchart in such cases. Journals should inform reviewers that their identity might be disclosed to their institution in cases of suspected misconduct.

Journals and publishers should retain peer review records to enable the investigation of peer review manipulation or other inappropriate behaviour by authors or reviewers.

#### III. Issues arising after misconduct investigations

##### Issue 13: institutions sometimes do not share findings of misconduct investigations with journals that have published affected research and journals may be reluctant to publish informative retraction notices

Journals have a duty to avoid misleading their readers and therefore sometimes need to correct or retract published work that is incorrect or unreliable. Since problems can arise either inadvertently, from honest error, or from deliberate misconduct, the reason for a retraction should be clearly stated in the retraction notice including details of the affected findings and the type of problem detected [18]. This is important to ensure that honest researchers are not discouraged from alerting journals to problems with their work because of fears that a retraction will damage their career or be taken to imply that misconduct has occurred (when, in fact, such honesty and care for the research record should be praised [19]). Journals that have published affected work therefore need to receive details of misconduct investigations including clear information about all of the published articles (and submitted manuscripts) that are affected.

Being able to quote or cite an official report from an institution should facilitate the publication of clear and informative retractions (or corrections) since it reduces the journal’s risk of litigation. If a journal reports that University X has investigated the case and determined that a researcher has fabricated data this is a statement of fact and therefore unlikely to expose the journal to claims that it has published defamatory material.

Although, after misconduct has been found, institutions often require researchers to contact journals in which their work was published, we encourage institutions also to contact the journals directly. This direct communication between institution and journal allows relevant information to be shared and avoids situations in which researchers fail to contact affected journals, refuse to accept an investigation’s findings, or give a misleading account of the investigation to the journal. If an author tells a journal that the investigation was unfair or its finding was incorrect, this places the journal in a difficult position, but this problem may be avoided if the journal is allowed to see relevant sections or the full report of the investigation and can therefore verify whether it was properly conducted. We also recommend that institutions should be transparent about their processes for handling suspected misconduct or, at least be prepared to share information about such processes with journals, if requested.

*Recommendations*

Institutions should notify journals directly and release all relevant sections of reports of misconduct investigations (or a summary of their findings) to all journals that have published research that was the subject of the investigation. Names may be redacted to ensure privacy.

Institutions should allow journals to quote from misconduct investigation reports or cite them in retraction statements and related publications (e.g. explanatory editorials or commentaries).

##### Issue 14: journals sometimes fail to respond to requests for correction or retraction from institutions or authors

Communication with a journal should normally be addressed to the editor, but if the editor does not respond, the publisher should be contacted. If a journal is owned by an academic society, the leaders of that society may also be used as a point of contact, or to raise concerns about the behaviour of the editor.

*Recommendation*

Journals should respond to institutions about research integrity cases in a timely manner. The editor should be the first point of contact, and journals should publish their contact details. If a journal editor does not respond, the publisher or journal owner should be contacted.

## Discussion

Research institutions and academic journals have important but different responsibilities with respect to the integrity of research and its reporting. Inaccurate, false or incomplete publications may mislead readers, waste research resources, and undermine the reliability of decisions based on them. It is therefore important that journals and institutions cooperate over cases in which there are concerns about the integrity of research reports that have been published or submitted for publication. However, such cases are rarely straightforward and may raise issues such as the ones described in this document and the earlier ones on which it is based [1, 2].

Many of the issues described in this document fall into the category of complex or ‘wicked’ problems lacking simple solutions and involving multiple stakeholders. While this document focuses on interactions between research institutions and academic journals, we recognise that the solutions to some of the problems raised will involve other organizations such as funders. We therefore hope they might be involved in future discussions. Another feature of ‘wicked’ problems is that they are difficult to define or categorize and each one is subtly different, so it is hard to produce guidance that is applicable in all cases. Unlike medical treatment guidelines, where the problem is clearly defined and evidence is usually available from scientific research, little or no research has been done in this area, so techniques such as systematic literature reviews are of little benefit. While methodology has been described for producing medical research reporting guidelines, this did not seem applicable to such a broad topic encompassing a wide range of problems and possible solutions.

Some of the problems tabulated at WCRI in Montreal arise because of conflicts in ethical principles or legal and regulatory frameworks. Thus, an institution may endeavour to uphold confidentiality in an investigation while a journal wishes to ensure transparency and avoid misleading its readers. Both are valid and laudable approaches, but they sometimes conflict and cause an impasse. We identified several situations in which parties, following relevant guidance or legislation, and trying to do the right thing, nevertheless create problems which may have serious consequences.

Institutions, journals and research funders should consider research integrity issues when formulating or reviewing their policies and practices. All have a responsibility for establishing, promoting and incentivising cultures that encourage the integrity of research and publications and of avoiding perverse incentives that inadvertently reward low quality research or misconduct [20].

### Topics needing further discussion / harmonization of guidelines

Laws, traditions and practices around the confidentiality of disciplinary hearings and misconduct investigations vary widely between countries and regions. As noted by ENRIO, in some countries cases are handled in strict confidence and decisions are not made public while in others, the public has the right to access decisions and even underlying documents [11]. Sharing information with journals may be hampered by guidelines which state that any disclosure to third parties should be made on a confidential basis [11]. However, it should be noted that the OECD guidelines state that ‘where possible’ such disclosure should be confidential and recognise that journals are one of the organizations (along with funders) to whom reports of misconduct investigations should be provided [21]. The CLUE Working Group included members from a wide range of countries and recognised that institutions are bound by local employment laws which may need to be revised or harmonized to facilitate the sharing of information between institutions and journals.

Another topic requiring further discussion are the circumstances under which journals should contact an institution directly before contacting the authors. While recommending that journals should develop criteria for determining this, we recognise that further guidance would be helpful. However, this is a difficult area. For example, Retraction Watch (a blog that reports on retractions) initially recommended raising research integrity concerns with the editor of the journal where the work was published, who would, in turn contact the authors, but later advised that the institution’s research integrity officer should be contacted first [22].

#### Next steps

We hope that these recommendations provide a useful starting point for further discussion. We encourage all research institutions, academic journals and funders to consider how they might implement the recommendations and thus improve future collaboration and cooperation. We also hope that organizations that represent and bring together research institutions at national and regional levels will initiate discussion on this topic.

#### Recommendations on best practice

##### Research institutions should

- have a research integrity officer (or office) and publish their contact details.
- publish their processes for conducting inquiries and investigating misconduct and share information about such processes with journals, on request.
- notify journals directly and release all relevant sections of reports of misconduct investigations (or a summary of their findings) to any journals that have published research that was the subject of the investigation, clearly indicating which articles or manuscripts are affected.
- allow journals to quote from misconduct investigation reports or cite them in retraction statements and related publications (e.g. explanatory editorials and commentaries).
- develop mechanisms for assessing the validity of research reports that are submitted to, or published by, academic journals, which could be used if concerns are raised; these mechanisms should be distinct from processes to determine whether misconduct has occurred.
- take responsibility for all research performed under their auspices regardless of whether the researcher still works at that institution or how long ago the work was done.
- be responsive to journal requests for information to ensure that peer reviewers’ and authors’ competing interests are properly disclosed.
- judge anonymous or pseudonymous allegations on their merit and not dismiss them automatically.
- ensure that essential research data are retained for at least 10 years (enabling the validation of published findings) and that responsibility (and resources) for data storage (e.g. for multicentre studies) is included in funding agreements.

##### Academic journals should

- publish the contact details of the Editor-in-Chief or a person with specific responsibility for handling research integrity concerns.
- respond to institutions about research integrity cases in a timely manner.
- have criteria for determining whether, and what type of, information and evidence relating to the integrity of research reports should be passed on to institutions.
- have criteria for determining when reviewer or editor suspicions that work is ‘too good to be true’ or that something is ‘not right’ should be shared.
- share evidence of misconduct by researchers acting as peer reviewers with their institution and follow the relevant COPE flowchart in such cases.
- explain to reviewers that their identity might be disclosed to their institution in cases of suspected misconduct and that serious misconduct cases will be addressed by the institution.
- have criteria to determine when the authors’ institution(s) should be contacted immediately without (or at the same time as) alerting the author(s). This would occur only in exceptional cases when a journal had evidence of substantive or significant falsification or fabrication of data.
- NOT reveal to institutions the identity of peer reviewers or other people raising concerns (unless this is already published or the individuals have given permission for this disclosure).
- seek explanations from authors about research integrity concerns even if they do not intend to accept their publication
- pass on research integrity concerns to institutions, regardless of whether they intend to accept the work for publication.
- judge anonymous or pseudonymous allegations on their merit and not dismiss them automatically.
- request corresponding authors from areas without a national (or regional) register of institutional research integrity officers, to provide the name and contact details of their institution’s research integrity officer (or of an individual with responsibility for handling research integrity allegations).
- retain peer review records for at least 10 years to enable the investigation of peer review manipulation or other inappropriate behaviour by authors or reviewers.

##### Funders should

- ensure that essential research data are retained for at least 10 years (enabling the validation of published findings) and that responsibility and resources for data storage (e.g. for multicentre studies) are included in funding agreements.
- be responsive to journal requests for information to ensure that peer reviewers’ and authors’ competing interests are properly disclosed.
- ensure that research agreements specify the primary institution (that will coordinate the response to any research integrity concerns).

National research integrity bodies (or other appropriate organizations, e.g. regional bodies) should keep a register of people with responsibility for handling research integrity allegations at their country’s institutions, to enable journal editors (and others) to contact them.

#### Issues requiring more complex solutions or further discussion

Journals need to be aware of differences in national and regional legal systems and institutional procedures to determine what details institutions can release about misconduct and disciplinary investigations. However, institutions also need to understand journals’ responsibilities for promptly alerting readers to potentially misleading publications (e.g. via expressions of concern).

## Supplementary Information

**Additional file 1.**

## Data Availability

Not applicable.
