# Supplementary material for: Cooperation & Liaison between Universities & Editors (CLUE): recommendations on best practice
Source: Res Integr Peer Rev. 2021 Apr 15;6:6. doi: 10.1186/s41073-021-00109-3 (PMC8048029; doi:10.1186/s41073-021-00109-3)
Supplement: Supplementary file 1 — Additional file 1. [file 41073_2021_109_MOESM1_ESM.docx]

**Collaboration & Liaison between Universities & Editors (CLUE)**

**Programme**

**Day 1: Monday July 11^th^ 2016**

*Evening:*

Working dinner

- Welcome
- Introductions
- Ground rules
- How we got here (very short recap / scene setting)
- Statement of work for the group / discussion of agenda
- Agree scope of topics / definition of misconduct

**Day 2: Tuesday, July 12th**

*Morning session (I)*

8.30-10.00

Institutions’ expectations of journals:

- suspected author / reviewer misconduct (I^1^)

Kick-off talk (5 min): View of a Research Integrity Officer

Coffee break (10.00-10.30)

*Morning session (II)*

10.30-12.00

Journals’ expectations of institutions:

- suspected author / reviewer misconduct (J^1^)

Kick-off talk (5 min): View of an Editor from a large journal

Lunch (12.00-13.00)

*Afternoon session (III)*(13.00-14.30)

Institutions’ expectations of journals:

- proven misconduct (I^2^)

Kick-off talk (5 min): View of a researcher

Coffee break (14.30-15.00)

*Afternoon session (IV)*(15.00-16.30)

Journals’ expectations of institutions:

- proven misconduct (J^2^)

Kick-off talk (5 min): View of an Editor from a smaller journal

**Day 2: Wednesday, July 13th**

*Morning session (V)*

(08.00-9.30)

Institutions’ expectations of journals:

- other situations (eg authorship disputes, retractions for honest error) (I^3^)

Kick-off talk (5 min): View of a senior academic

Coffee break (9.30-9.50)

*Morning session (VI)*(9.50-11.20)
Journals’ expectations of institutions:

- other situations (eg authorship disputes, retractions for honest error) (J^3^)

Kick-off talk (5 min): View of a journal publisher

Short break 11.20-11.30

*Morning session (V11)*

(11.30-12.30)

Other stakeholders (funders, ORI, journalists, lawyers) (O)

Kick-off talk (5 min): View of a lawyer involved in research integrity cases

Lunch (12.30-13.00)

*Afternoon session (VIII)*

(13.00-14.30)

Finalize / discuss wording

Coffee break (14.30-14.50)

*Afternoon session (IX)*

*(2.50-15.30)*

Next steps

Wrap-up, charge to group for further work, reporting out

*Themes / topics*

Institutions’ expectations of journals:

- suspected author / reviewer misconduct (I^1^)

- proven misconduct (I^2^)

- other situations (eg authorship disputes, retractions for honest error) (I^3^)

Journals’ expectations of institutions:

- suspected author / reviewer misconduct (J^1^)

- proven misconduct (J^2^)

- other situations (eg authorship disputes, retractions for honest error) (J^3^)

Other stakeholders (funders, ORI, journalists, lawyers) O

Each session to have focused discussion based on questions outlined in Montreal document (to be circulated in advance). First speakers in I sessions will be representatives of institutions. First speakers in J sessions will be representatives of journals.

However, we won’t invite formal talks, but ask somebody to give a 5 minute kick-off talk to get the discussion going, highlighting the areas they think cause the most problems from their viewpoint.

*Running times:*

- Sessions: 1.5 hours
- Breaks: 30 minutes (last break 15 minutes)
- Lunch Day 2: 1 hour
- Lunch Day 3: 30 minutes (sandwich lunch – shorter break to allow early finish)
